# Is There a Body Mass Index Threshold for Patients Undergoing Primary Total Knee Replacement—A Literature Review

**DOI:** 10.3390/jcm15010103

**Published:** 2025-12-23

**Authors:** Muhammad Kamran, Mahmoud Abumarzouq, Anant Mahapatra

**Affiliations:** 1 Senior House Officer Orthopaedics & Trauma Surgery, OLHN, MBBS, C15 RK7Y Navan, Ireland; 2 Registrar Orthopedics & Trauma Surgery, OLHN, C15 RK7Y Navan, Ireland; mahmoudabumarzouq@gmail.com; 3 Consultant Orthopedics & Trauma Surgery, OLHN, OLOL Drogheda Ms (Orth), FRCS, MCh (Orth), FRCS (Orth), FFSEM, FIOA, C15 RK7Y Navan, Ireland; mahapatra.anant@gmail.com

**Keywords:** osteoarthritis, total knee replacement, body mass index, obesity, surgical outcomes, postoperative complications, patient selection, functional recovery

## Abstract

**Background:** Osteoarthritis (OA) is a prevalent degenerative joint disease and a major cause of disability in the aging population. Total knee arthroplasty (TKA) is a common intervention for advanced OA, yet postoperative outcomes may vary, particularly among individuals with obesity. Elevated body mass index (BMI) is a recognized risk factor for the development and progression of OA and may influence perioperative and postoperative complication rates. **Objective:** This literature review evaluates whether a specific BMI threshold should guide eligibility for primary TKA, with particular emphasis on the impact of BMI on surgical risk, implant outcomes, and functional recovery. **Methods:** A systematic search was conducted across PubMed (MEDLINE), Cochrane Library, EMBASE, and Google Scholar to identify peer-reviewed studies from the past two decades examining the relationship between BMI and clinical outcomes following primary TKA. **Findings:** Higher BMI—especially ≥40 kg/m^2^—is consistently associated with increased perioperative and postoperative complications, including wound issues, infection, thromboembolic events, longer hospital stay, and higher revision risk. Despite these elevated risks, evidence demonstrates that obese and morbidly obese patients experience substantial improvements in pain, mobility, and function that are comparable in magnitude to those seen in non-obese individuals. The literature does not support a universally applicable BMI cutoff for determining surgical eligibility. **Conclusions:** BMI is an important modifier of surgical risk but should not be used as an absolute criterion for excluding patients from TKA. Instead, a personalized approach is recommended—one that considers BMI within the context of comorbidities, functional limitation, patient motivation, and opportunities for preoperative optimization. With appropriate patient selection and risk-mitigation strategies, TKA remains a clinically valuable and justified intervention across all BMI categories.

## 1. Introduction

### 1.1. Context of the Problem

Osteoarthritis (OA) is a leading cause of pain, disability, and loss of mobility in older adults, with rising incidence due to population ageing and obesity. Total knee replacement (TKR) is the most common surgical treatment for end-stage knee OA and the second most frequently performed procedure in people over 60 years of age in the UK, with over 63,000 procedures performed annually. Although TKR is generally effective in improving pain and quality of life, up to 20% of patients report unexpected or suboptimal postoperative outcomes [1].

Articular cartilage (AC) is an avascular tissue that enables smooth joint movement and load bearing. Its limited capacity for repair means cartilage damage often progresses to OA, resulting in pain and functional impairment. While joint replacement is an effective treatment, it remains invasive and associated with perioperative risk [2].

Obesity is a major contributor to knee pain and disability and is increasingly prevalent among patients undergoing TKR. Elevated BMI increases mechanical loading of the knee and is independently associated with worse pain and reduced function. Webb et al. reported a three- to four-fold increased risk of knee pain with disability in obese individuals, with 36% of cases attributable to elevated BMI [3].

Despite this, the influence of BMI on postoperative outcomes following TKR remains debated. While higher BMI is linked to increased complication risk, many obese patients still achieve meaningful functional improvement. This review therefore examines BMI-related outcomes following primary TKR and evaluates whether a clinically relevant BMI threshold should inform surgical decision-making.

### 1.2. Purpose of the Review

- To explore whether there is an optimal BMI threshold for performing TKA.
- To evaluate how BMI affects surgical outcomes, complications, and decision-making processes for TKA.

### 1.3. Research Question

- Is there a specific BMI threshold that should influence the eligibility of patients for primary TKA?

## 2. Methodology

### 2.1. Study Design

This study was conducted as a focused literature review addressing the research question:

“Is there a body mass index (BMI) threshold associated with postoperative outcomes in patients undergoing primary total knee replacement (TKR)?”

Although not a systematic review, the search, screening, and reporting processes were structured in accordance with PRISMA 2020 principles to enhance transparency and methodological rigor [4].

Two categories of literature were included:

1. Primary studies (*n* = 6): Original clinical studies reporting quantitative associations between BMI (continuous or categorical) and postoperative TKR outcomes, including complications, length of stay, functional outcomes, or revision risk.
2. Supporting secondary sources: Review articles, guidelines, and related literature used to contextualize findings. These sources were not included in the PRISMA flow diagram or primary synthesis.

### 2.2. Eligibility Criteria

#### 2.2.1. Inclusion Criteria (Primary Studies)

Studies were included if they met all of the following criteria:

- Study type: Original clinical research (cohort, case–control, comparative studies, or national registry/database analyses).
- Population: Adults (≥18 years) undergoing primary TKR; mixed total joint arthroplasty datasets were included only when TKR-specific BMI data were extractable.
- Exposure: BMI reported as a continuous variable, categorical classification, or defined threshold.
- Outcomes: Postoperative clinical outcomes following TKR, including complications, length of stay, functional outcomes, or revision risk.
- Publication period: January 2013 to December 2023.
- Language: English.

#### 2.2.2. Exclusion Criteria

Studies were excluded if they:

- Were reviews, scoping reviews, guidelines, commentaries, or expert opinions.
- Were biomechanical, imaging-based, or non-clinical studies.
- Did not report BMI-related postoperative outcomes.
- Focused on revision TKR or unicompartmental knee arthroplasty.
- Contained duplicate or non-primary data.

Secondary sources not meeting primary study criteria were retained solely for narrative support and were not included in PRISMA counts.

### 2.3. Information Sources

A structured literature search was conducted in the following databases:

- PubMed (MEDLINE).
- Cochrane Library.
- Google Scholar.

The final search was completed on 15 December 2023.

### 2.4. Search Strategy

Both MeSH terms and free-text keywords were used. The core PubMed search strategy was:

(“Body Mass Index”[MeSH] OR BMI OR obesity) AND
(“Total Knee Replacement” OR “Total Knee Arthroplasty”) AND
(outcomes OR complications OR thresholds)

Search strategies were adapted for each database.

### 2.5. Study Selection

All retrieved records were imported into Zotero for duplicate removal and screening.

- Title and abstract screening: Conducted independently by two reviewers. Discrepancies were resolved through discussion.
- Full-text review: Articles were assessed against predefined eligibility criteria. Studies lacking postoperative BMI-related outcomes or original clinical data were excluded as primary studies but retained as supporting references where relevant.

### 2.6. PRISMA-Informed Study Selection

A total of 2143 records were identified. After removal of 649 duplicates, 1494 records underwent title and abstract screening, resulting in 1312 exclusions. 

182 full-text articles were assessed, of which 176 were excluded for not meeting inclusion criteria.

✔ Primary studies included in final synthesis: 6

The limited number of eligible studies reflects the narrow postoperative focus of the research question and the scarcity of studies reporting explicit BMI thresholds linked to postoperative TKR outcomes.

## 3. PRISMA Flow Diagram (Literature Review Using PRISMA Principles)


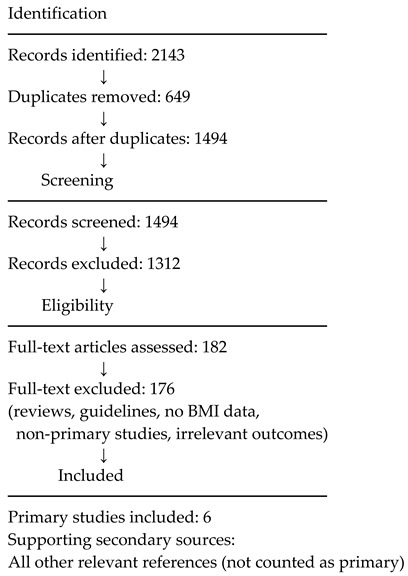


### 3.1. Data Collection

For the six included primary studies, data extraction was independently performed by two reviewers using a standardized form. Extracted data included:

- Study design and setting.
- Sample size.
- BMI values, categories, or thresholds.
- Postoperative outcomes (complications, length of stay, functional outcomes, revision risk).
- Key findings related to BMI and TKR outcomes.

### 3.2. Quality Assessment

Methodological quality was assessed using the **Newcastle–Ottawa Scale (NOS)** appropriate to each study design.

- Scores of **7–9** indicated high quality.
- Scores of **5–6** indicated moderate quality.

No studies were excluded based on quality; however, study quality was considered during interpretation of findings.

## 4. Relationship Between BMI and Knee Osteoarthritis

- **Link Between Obesity and OA:**
  Osteoarthritis is a multifactorial degenerative joint disease characterized by progressive loss of articular cartilage, subchondral bone remodeling, and synovial inflammation. Both biochemical and biomechanical factors influence its onset and progression. While physiological mechanical loading is essential for maintaining cartilage homeostasis, excessive loading—particularly in individuals with elevated BMI—accelerates cartilage breakdown and increases the risk of knee OA. Obesity contributes not only through mechanical overload but also through systemic pro-inflammatory mediators that may potentiate cartilage degradation and chronic joint inflammation [5].
- In longitudinal population-based cohorts, BMI has consistently been shown to be a strong, dose-dependent predictor of incident knee OA. One 10-year follow-up study of individuals without baseline OA or rheumatoid arthritis demonstrated that each higher BMI category significantly increased the likelihood of developing knee OA across all analytical models, confirming obesity as one of the strongest modifiable risk factors for this disease [6].
- **Increased Risk of Early-Onset OA in Individuals With High BMI:**
  Elevated BMI is also associated with earlier need for surgical intervention. Obesity increases the mechanical load across the tibiofemoral joint, accelerates degenerative changes, and shortens the time from symptom onset to severe functional limitation. Evidence shows that each unit increase in BMI reduces the age at primary TKA by approximately 0.56 years. Class III obesity is particularly impactful, with affected patients undergoing TKA on average **12.2 years earlier** than individuals with normal BMI. Similar trends have been observed for total hip arthroplasty (THA), highlighting obesity as a risk factor for accelerated joint failure [7].

## 5. Impact of BMI on Surgical Outcomes for Primary TKA

### 5.1. Short-Term Surgical Outcomes and Complications

- **Intraoperative Considerations:**
  Obesity has implications for perioperative risk, primarily due to comorbidities such as obstructive sleep apnea, metabolic syndrome, and cardiopulmonary limitations. Proper preoperative optimization and coordination with an experienced anesthesia team are essential; however, extensive descriptions of anesthetic techniques are beyond the scope of this review and were therefore streamlined to maintain focus on BMI as a determinant of surgical outcomes [8].
- **Postoperative Complications:**
  Higher BMI is consistently associated with increased postoperative complication rates following TKA. Obesity is a well-established risk factor for both superficial and deep periprosthetic joint infection. Reported infection rates range from approximately **0.9% to 2.19%**, with obese patients demonstrating significantly higher rates of early (<3 months) and late infections. These complications often necessitate surgical debridement and increase the likelihood of revision procedures. A synthesis of 20 observational studies identified markedly higher risks of infection, wound complications, and early postoperative morbidity among obese patients compared to those with normal BMI [9,10].
- **Hospital Stay and Readmission:**
  Higher BMI also correlates with increased resource utilization. A 2017 cohort analysis demonstrated a clear dose–response relationship between BMI category and length of stay (LOS). Compared with patients with BMI <25, LOS increased by **0.32, 0.33, 0.67, and 1.15 days** for overweight, class I, class II, and class III obesity, respectively. Class III obesity additionally doubled the odds of requiring facility-based discharge. Poorer preoperative physical function further prolonged hospitalization, indicating that both obesity and baseline functional limitation contribute to postoperative recovery trajectories [11].

### 5.2. Long-Term Outcomes: Implant Survival and Functional Improvement

- **Implant Longevity:**
  Evidence indicates that elevated BMI may negatively influence implant survival. A large cohort study by Prohaska et al. [11] reported that higher postoperative BMI was significantly associated with increased rates of reoperation and revision (*p* < 0.001). Patients with BMI ≥ 35 kg/m^2^ had substantially higher risks of wound complications (HR 1.07), deep infection (HR 1.08), and aseptic loosening or polyethylene wear. These findings suggest that mechanical overload and altered joint biomechanics contribute to accelerated implant failure. Importantly, BMI was not correlated with rates of venous thromboembolism, tibiofemoral instability, or manipulation under anesthesia [12].
- **Functional Outcomes:**
  Beyond pain alleviation, patients with obesity undergoing total knee arthroplasty demonstrate substantial postoperative improvements in functional outcomes. Evidence from multiple studies indicates that patient-reported outcome measures improve across all body mass index categories, with obese individuals achieving comparable relative gains in pain reduction, functional performance, and health-related quality of life after TKA. These findings suggest that, although preoperative functional status may differ, clinically meaningful functional recovery is attainable in patients with obesity, supporting total knee arthroplasty as an effective therapeutic intervention in this population. [13].
- Overall, current evidence suggests that elevated BMI is associated with increased perioperative risks and potentially reduced implant longevity, but **does not preclude patients from achieving substantial functional gains** after TKA.

## 6. Is There a BMI Threshold for TKA?

### 6.1. Debate on BMI as an Absolute Contraindication

The question of whether a BMI threshold should limit access to primary TKA remains contentious. Contemporary studies highlight the limitations of BMI as a sole predictor of surgical risk. JD et al. note that improvements in implant design and perioperative management have enhanced outcomes for patients with higher body mass, but emphasize that BMI alone does not reliably predict implant failure. They advocate for clearer guidelines regarding implant load tolerances and for thorough surgeon–patient discussions to ensure informed consent and mitigate medicolegal risk [14].

Some evidence suggests that higher BMI does not necessarily lead to inferior implant outcomes. One study reported no significant differences in implant survival or revision rates among patients with elevated BMI. Notably, individuals with BMI ≥ 35 kg/m^2^ experienced the greatest functional improvements after unicompartmental knee arthroplasty (UKA). When appropriately indicated, the authors argued that high BMI should not constitute a contraindication and that imposing a fixed BMI threshold—such as 35 kg/m^2^—may be unjustified [15].

#### 6.1.1. Arguments Supporting a BMI Threshold

Proponents of BMI cutoffs—often citing values around 40 kg/m^2^—refer to the association between increasing BMI and higher perioperative complication rates. One study reported that median BMI increased significantly postoperatively (from 28.68 to 30.75 kg/m^2^), underscoring the long-term difficulty of weight management following arthroplasty and highlighting the need for preoperative optimization [16].

Institutional and hospital guidelines frequently recommend delaying surgery in patients with BMI > 40. Wallace et al. observed that obese patients were at increased risk for wound infection and thromboembolic events. However, while risks rose with BMI, absolute complication rates remained low. The authors emphasized that BMI should inform patient counseling but should not serve as the sole criterion for denying surgery, noting that carefully selected high-BMI patients can still achieve excellent outcomes [17].

Large registry-based studies further demonstrate a dose-dependent increase in perioperative risk. In an analysis of 268,663 patients, those with BMI > 30 kg/m^2^ experienced higher rates of infectious and medical complications than non-obese individuals. Patients with BMI > 40 kg/m^2^ had longer operative times, greater hospital resource utilization, and increased risks of readmission, reoperation, deep vein thrombosis, renal complications, and postoperative infection. Similar trends were observed across obesity classes I–III [18].

#### 6.1.2. Arguments Against a Strict BMI Threshold

Critics caution that rigid BMI cutoffs may unnecessarily restrict access to a procedure that offers substantial symptomatic and functional benefit. One study found that fewer than 1 in 20 TKA patients required revision within 10 years, with slightly higher but still acceptable revision rates in obesity classes I and II [19]. Ninety-day mortality was rare across all groups, with the highest risk found in underweight—not obese—patients.

Though overweight and obese individuals may experience marginally less functional improvement, evidence consistently shows meaningful postoperative gains. Li et al. [20] reported that severely and morbidly obese patients achieved excellent pain relief and substantial functional improvement six months after total joint replacement, comparable to patients with lower BMI. While obesity increases the risk of early postoperative complications, these findings suggest that it should not be viewed as an absolute barrier to TKA, given its clear potential to improve quality of life [20].

### 6.2. Surgical Guidelines and Recommendations

#### 6.2.1. Current Practices and Guidelines

National clinical guidelines differ regarding BMI and surgical eligibility.The American Academy of Orthopaedic Surgeons (AAOS) states that obesity elevates the risk of perioperative complications—such as infection, poor wound healing, thromboembolism, respiratory complications, and higher readmission rates—and cites BMI < 40 as the generally accepted threshold for elective joint replacement [21].

In contrast, the National Institute for Health and Care Excellence (NICE) does **not** recommend restricting access to TKA based on BMI, age, or sex, advocating instead for equitable access to care [22].

Expert opinion similarly supports individualized evaluation. Dr. Nicholas A. Bedard of the Mayo Clinic emphasizes that recommending TKA or THA in patients with BMI ≥40 requires careful, personalized assessment of operative risk weighed against expected functional benefit [23].

#### 6.2.2. Individualized Approach to Patient Selection

Beyond BMI, patient-specific factors—including comorbidity burden, nutritional status, mobility, psychological readiness, and expectation alignment—significantly influence postoperative outcomes. In a cohort of 1374 knee OA patients, 78.1% experienced positive outcomes one year after TKA, despite a 2.2% complication rate. Greater preoperative symptom severity, lower depression levels, strong commitment to surgery, and realistic expectations were associated with improved outcomes [24].

Shared decision-making (SDM) is integral to optimizing patient selection and satisfaction. Studies show that SDM improves patient understanding, enhances satisfaction, and can be incorporated into routine consultations without extending visit length. Decision aids further increase patient knowledge, help align treatment with personal values, reduce unnecessary surgery, and promote adherence to postoperative rehabilitation. Including family members in the process can further reinforce engagement and compliance [25].

## 7. Role of Preoperative Weight Loss in Improving Outcomes

### 7.1. Evidence Supporting Weight Loss Before TKA

Emerging evidence suggests that preoperative weight reduction may diminish postoperative complications. A systematic review reported that weight loss—measured by BMI reduction—may decrease or have no impact on postoperative complication rates, though the strength of evidence remains limited. More randomized controlled trials are needed to determine whether weight loss directly improves clinical outcomes or whether associated behavioral changes (e.g., improved nutrition, reduced inflammation) contribute more substantially. Existing guidelines may also require revision, as overly aggressive weight reduction goals may place some patients at risk [26].

### 7.2. Challenges in Achieving Preoperative Weight Loss

Patients with knee OA frequently struggle to lose weight due to chronic pain, limited mobility, inflammation-related fatigue, and reduced ability to exercise. Current recommendations advise all obese OA patients to aim for approximately **10% body weight loss**, though achieving and sustaining this reduction remains difficult. Ongoing research seeks to clarify the benefits of sustained weight loss on OA progression [27].

## 8. Results

### 8.1. Study Selection

The search yielded **2143 records**, from which **649** duplicates were removed, leaving **1494** articles for title and abstract screening. Of these, **1312** were excluded as irrelevant. A total of **182** full-text articles were assessed, and **176** were excluded for not providing original BMI-related postoperative outcomes.

Ultimately, **six primary studies** were included in the final evidence synthesis. All other relevant references were retained as **supporting literature** but were not counted as primary studies.

### 8.2. Characteristics of Included Studies

The six included primary studies consisted of prospective and retrospective cohorts and large national database analyses. Sample sizes ranged from **90** to **268,663** patients. BMI was consistently reported either in WHO categories or as clinically meaningful thresholds (e.g., **BMI ≥ 30**, **≥35**, **≥40**).

A full summary of included studies is presented in Table 1.

## 9. Association Between BMI and Surgical Outcomes

Across all primary studies and supporting evidence, **higher BMI was linked with increased perioperative and postoperative complications** following TKA.

### 9.1. Prosthesis Survival and Longevity

Higher BMI, especially **BMI ≥ 40**, was associated with:

- Increased tibial component loosening.
- Increased polyethylene wear.
- Higher mechanical failure rates.
- Greater need for revision surgery.

One cohort found that **morbidly obese patients had significantly higher revision rates** than non-obese patients (*p* = 0.02) [28].

### 9.2. Wound Complications

Obese patients experienced higher rates of:

- Wound separation.
- Delayed healing.
- Superficial wound infection.

These findings reflect poorer vascularity and increased soft tissue tension.

### 9.3. Deep Prosthetic Joint Infections (PJI)

BMI ≥ 40 was consistently associated with:

- Higher incidence of deep infection.
- Increased need for surgical debridement.
- Higher progression to revision arthroplasty.

### 9.4. Intraoperative Challenges

Increased adiposity contributed to:

- Difficult exposure.
- Longer operative times.
- Increased intraoperative blood loss [29].

## 10. Functional and Patient-Reported Outcomes

Despite the increased complication risks, obese and morbidly obese patients showed **meaningful clinical benefit** from TKA.

### 10.1. Pain Reduction

Patients with **BMI ≥ 30** demonstrated substantial:

- Pain relief.
- Improved walking tolerance.
- Improved quality of life.

### 10.2. Functional Gains and Satisfaction

Although regaining full knee flexion can be slower:

- Postoperative functional improvement was consistent across BMI categories.
- Satisfaction rates remained high.
- Patients frequently reported a marked reduction in disability.

### 10.3. Comparative Functional Outcomes

One U.S. study reported:

- **88%** of obese patients achieved a **Knee Society Score ≥ 80** at 80 months.
- **99%** of non-obese patients achieved the same.
- Morbidly obese individuals had higher revision rates (*p* = 0.02) [30].

Yet functional improvement was similar in magnitude across all BMI groups.

## 11. Surgical Considerations and Innovations

Current advancements focus on complication minimisation rather than exclusion based on BMI.

### 11.1. Enhanced Recovery and Infection Prevention

Recent improvements include:

- ERAS protocols.
- Optimised infection prevention strategies.
- Preoperative optimisation clinics.

### 11.2. Technical Modifications for Obese Patients

Recommended adjustments include:

- Short-stem augments for improved tibial load sharing.
- Extended incisions for safer exposure.
- Multi-layered barbed sutures.
- Bipolar sealants.
- Negative pressure wound therapy.

These strategies aim to reduce wound-related risks and improve recovery consistency among obese patients [31].

## 12. Synthesis of BMI Threshold Evidence

Across all six primary studies:

**BMI ≥ 40**

- **Strong and consistent evidence** of significantly increased risk.
- Highest rates of infection, wound problems, VTE, revision surgery, and prolonged LOS.

**BMI 35–39.9**

- Moderately increased risk.
- Some complications show a stepwise rise from Class I → II → III.

**BMI 30–34.9**

- Small risk increase.
- **No meaningful negative impact on functional improvement.**

**Overall Interpretation**

- Obesity is associated with increased complications, especially at **BMI ≥ 40**.
- Nevertheless, obese patients experience **similar functional gains** and **significant pain reduction**, making TKA clinically beneficial across all BMI categories (Table 2).

## 13. Discussion

This review demonstrates a consistent association between increasing BMI and a higher risk of perioperative and early postoperative complications following total knee arthroplasty (TKA). Across the six included primary studies, elevated BMI—particularly values ≥40 kg/m^2^—was associated with increased rates of wound complications, deep infection, venous thromboembolism, prolonged operative time, and early readmission or reoperation. These findings are biologically plausible and reflect the combined mechanical, metabolic, and inflammatory challenges posed by severe obesity during and after surgery.

However, a critical interpretation of the evidence highlights that while complication rates increase with BMI, functional benefit from TKA is largely preserved across BMI categories. Several studies reported comparable improvements in pain relief, mobility, and patient-reported outcome measures among obese and non-obese patients. This suggests that obesity primarily influences surgical risk rather than treatment efficacy, an important distinction when considering access to surgery. Notably, none of the included studies demonstrated a BMI threshold beyond which functional outcomes were uniformly poor, challenging the justification for rigid BMI-based exclusion criteria.

At the same time, the evidence base has important limitations. Outcome definitions, BMI categorisation, and follow-up durations varied substantially across studies, limiting direct comparison and precluding meta-analysis. Many studies relied on large registry or database designs, which, while powerful for detecting complications, may underrepresent patient-reported outcomes and longer-term functional recovery. Additionally, BMI alone is an imperfect surrogate for surgical risk, as it does not account for body composition, fat distribution, or obesity-related comorbidities that may independently influence outcomes.

Taken together, these findings support a risk-stratified, patient-centred approach rather than the use of absolute BMI thresholds. While higher BMI is clearly associated with increased perioperative risk, the consistent functional gains observed following TKA indicate that BMI alone should not be used to deny surgery. Instead, careful patient counselling and targeted perioperative optimisation are likely to offer greater benefit than blanket exclusion policies.

### 13.1. Clinical Implications

The findings of this review suggest several strategies to improve outcomes in patients with elevated BMI undergoing TKA.

Preoperative optimisation remains central. Interventions such as weight reduction where achievable, glycaemic control, smoking cessation, nutritional support, and optimisation of comorbidities may reduce complication risk. Importantly, evidence suggests that even modest improvements in metabolic health, rather than large BMI reductions, may positively influence postoperative recovery.

From an intraoperative perspective, technical adaptations may mitigate some obesity-related challenges. These include improved surgical exposure, enhanced soft-tissue handling, layered wound closure techniques, and the use of adjunctive measures such as negative-pressure wound therapy. While such strategies are widely recommended, high-quality comparative data evaluating their effectiveness specifically in obese populations remain limited.

Postoperative management should prioritise vigilant wound surveillance, early mobilisation, extended thromboembolism prophylaxis, and structured rehabilitation. Enhanced Recovery After Surgery (ERAS) pathways may be particularly valuable in this group, although further evaluation of their effectiveness in severely obese patients is warranted.

### 13.2. Future Research Directions

The limited number of eligible studies identified in this review underscores a significant gap in the literature. Future research should focus on:

- Defining clinically meaningful BMI or risk thresholds that integrate metabolic health and comorbidity burden rather than BMI alone.
- Assessing long-term implant survival and revision risk across stratified BMI groups.
- Evaluating the effectiveness of structured preoperative optimisation or weight-management programmes.
- Investigating contemporary implant designs and surgical techniques that may better accommodate increased joint loading.

Addressing these gaps would support more nuanced clinical decision-making and help move beyond simplistic BMI-based eligibility criteria for TKA.

## 14. Conclusions

This review demonstrates that body mass index is a significant modifier of perioperative and postoperative risk following total knee arthroplasty (TKA). Higher BMI—particularly values ≥ 40 kg/m^2^—is consistently associated with increased rates of complications, including wound problems, infection, venous thromboembolism, prolonged hospital stay, and early revision. Importantly, however, obese and morbidly obese patients continue to experience substantial and clinically meaningful improvements in pain, mobility, and functional outcomes following TKA, comparable to those observed in patients with lower BMI.

The current evidence does not support a universally applicable BMI threshold for determining eligibility for TKA. While BMI cut-offs are frequently used to guide risk stratification and preoperative counselling, rigid exclusion based on BMI alone is not justified. Instead, surgical decision-making should adopt an individualised approach that considers BMI in conjunction with comorbidity burden, functional impairment, and patient-specific goals.

Optimisation strategies play a central role in improving outcomes for patients with elevated BMI. Preoperative risk modification, tailored intraoperative techniques, and structured postoperative recovery pathways can mitigate complication risk and support safe surgical care. Excluding patients from TKA solely on the basis of BMI risks denying effective treatment to individuals who may achieve significant quality-of-life benefits.

In conclusion, although elevated BMI increases surgical risk, TKA remains a valid and beneficial intervention for appropriately selected and optimised patients across all BMI categories. Future research should prioritise refined risk stratification beyond BMI alone, evaluate targeted optimisation programmes for severe obesity, and assess long-term implant outcomes to better inform evidence-based clinical decision-making.

## Figures and Tables

**Table 1:** Characteristics of Included Primary Studies (*n* = 6).

No.	Study (Author, Year)	Country	Study Design	Sample Size	BMI Measures	BMI Thresholds/Categories	Key Outcomes/Findings
1	Grotle et al., 2008 [6]	Norway	Prospective cohort (10-year follow-up)	1675	Mean BMI 24.2	BMI ≥30	Obesity increased 10-year knee OA risk (OR 2.81).
2	Prohaska et al., 2017 [11]	USA	Prospective consecutive cohort	716	WHO BMI classes	<25, 25–29.9, 30–34.9, 35–39.9, ≥40	Higher BMI → ↑LOS + ↑rehab discharge. BMI ≥ 40 doubled rehab transfer.
3	Li et al., 2017 [20]	USA	Prospective national cohort (FORCE)	2964	WHO BMI classes	Same as above	All BMI groups improved similarly; morbidly obese had greatest pain relief.
4	Barahona et al., 2023 [16]	CHILE	Observational Cohort	90	Median BMI 28.68	Age-adjusted BMI groups	BMI increased post-TKA (+2.07); PROMs unchanged across BMI groups.
5	JBJS Obese Cohort	USA	Retrospective matched cohort	146	Obesity = BMI ≥ 30	≥30; morbid obesity subgroup	Obese had lower KSS success; morbid obesity → ↑revision rate (*p* = 0.02).
6	NSQIP BMI-Stratified Study	USA	Retrospective national database	268,663	Continuous & categorical BMI	30–34.9; 35–39.9; ≥40	Stepwise ↑complications; BMI ≥ 40 highest risk of infection, DVT, reoperation.

**Table 2:** Newcastle–Ottawa Scale (NOS) Quality Assessment. Symbols ★★, ★★★, ★★★★ in the table are just for the clarity of rating.

No.	Study	Selection	Comparability	Outcome	Total	Quality Rating
1	Grotle et al., 2008 [6]	★★★★	★★	★★★	9/9	High
2	Prohaska et al., 2017 [11]	★★★★	★★	★★★	9/9	High
3	Li et al., 2017 [20]	★★★★	★★★	★★★	9/9	High
4	Barahona et al., 2023 [16]	★★★	★★	★★	7/9	High
5	JBJS Obese Cohort	★★★★	★★	★★	8/9	High
6	NSQIP BMI-Stratified Study	★★★★	★★★	★★★	9/9	High

## Data Availability

The data used in this study are publicly available and can be accessed via PubMed; all data sources are cited within the article.
